# Population dynamics and identification of efficient strains of *Azospirillum* in maize ecosystems of Bihar (India)

**DOI:** 10.1007/s13205-011-0031-7

**Published:** 2011-10-21

**Authors:** Rinkee Verma, S. K. Chourasia, M. N. Jha

**Affiliations:** Department of Microbiology, Faculty of Basic Sciences and Humanities, Rajendra Agricultural University, Pusa, Samastipur, Bihar 848 125 India

**Keywords:** *Azospirillum*, Soil tillage, Soil type, Organic carbon, Biofertilizer

## Abstract

Information on inoculum load and diversity of native microbial community is an important prerequisite for crop management of microbial origin. *Azospirillum* has a proven role in benefiting the maize (*Zea mays*) crop in terms of nutrient (nitrogen) supply as well as plant growth enhancement. Bihar state has highest average national maize productivity although fertilizer consumption is minimum, indicating richness of *Azospirillum* both in terms of population and diversity in soils. An experiment was planned to generate basic information on *Azospirillum* population variation in maize soils under different agricultural practices and soil types of Bihar, to identify suitable agricultural practices supporting the target microorganism and efficient *Azospirillum* strain(s). No tillage, growing traditional maize cultivar, land use history (diara soil having history of maize cultivation), soil organic carbon (>1%) and intercrop with oat supported prevalence of *Azospirillum* in maize rhizosphere. Native *Azospirillum* population varied from 1 million to 1 billion/g soil under diverse agricultural practices and soil types. Such richness, however, does not necessarily mean that artificial inoculation of *Azospirillum* is not required in Bihar soils as 92% of *Azospirillum* isolates (50 isolates) were poor in nitrogen-fixing ability and 88% were poor on IAA production. Efficient strains of *Azospirillum* based on growth (three), acetylene reduction assay (three), IAA production (three), broad range of pH (two) and temperature tolerance were identified. The findings suggested that maize crop in Bihar should be inoculated in universal mode rather than site-specific mode.

## Introduction

Microbes offer an eco-friendly source of nutrients supply and could augment crop production, sustain soil health and minimize use of chemical fertilizers (Vence 2001; Jha and Prasad 2006; Franche et al. 2009). Thus, microbial diversity and richness are key inputs to our understanding of the role, function and significance of microorganisms in plant nutrient supply. Among microbial pool, *Azospirillum* seems to be an ideal candidate for maize due to endophytic nature (Bashan et al. 2004), nitrogen-fixing ability, IAA production (Doberneir and Pedrosa 1987) and ubiquitous occurrence (Huergo et al. 2008).

*Azospirillum* colonize maize irrespective of the soil or geographic location and can be considered as a dominant maize rhizosphere micro-flora. It was found in association with maize in both culture-based and culture-independent approaches (Roesch et al. 2008). Attachment to the root system is mediated by the polar flagellum and is followed by irreversible anchoring of the bacteria (Steenhoudt and Vanderleyden 2000). The plasmid-borne gene mediates colonization process. They colonise the rhizoplane and are found in high numbers upon emergence of lateral roots and also near the root cap (De Oliveira et al. 2002). Their preponderance and colonization vary with various factors such as soil types, fertilizer application, soil moisture content, crop rotation etc. (Tripathi et al. 2002; Aertsen and Michiels 2004; Bashan et al. 2004; Garbeva et al. 2008). *Azospirillum* inoculation to maize reduced the use of chemical fertilizers by 15–50% and increased yield by 5–30% (Fages and Arsac 1991; Hungria et al. 2010).

Maize has become major crop of small and marginal farmers, the predominant farming community of Bihar (India). It is now continuously cropped throughout the year and in all agro-climatic zones. This necessitated exploring possibility of maximum utilization of *Azospirillum* for cheap and safe nutrient sources for maize. This can be achieved either through artificial inoculation or identifying agricultural practices favoring its proliferation in maize ecosystem. Various studies indicated that the magnitude of *Azospirillum* responses varies with location, season, product quality etc. (Bashan et al. 2004). Further, chances of survival of alien strain is poor than autochthonous strains (Bashan et al. 1995). Thus, selection of region specific promising strain is the prerequisite for deriving maximum benefit from *Azospirillum.* In the present investigation, we focused on the assessment and identifying of native *Azospirillum* supportive maize cultivation systems and on selection of autochthonous efficient *Azospirillum* strains for use as an inoculants in future.

## Methods

### Site description and selection

The study was conducted in farmers’ maize fields in different soil types viz. Vertisol (tal), Entisol/Inceptisol (diara, calcareous, non-calcareous) under different agricultural practices viz*.* tillage system, crop varieties, history of cropping pattern, cropping systems (monocropping, intercropping etc.). The physico-chemical properties of ‘diara’ soil and ‘tal’ soils (Table 1) revealed that the water-holding capacity of ‘diara’ soil is remarkably low than ‘tal’ soils. These soils are mostly low in organic carbon contents due to poor vegetation and high rate of organic matter decomposition under the hyperthermic region. The soils are generally alkaline in reaction. The ‘diara’ soils are low in major and secondary nutrients. The available micronutrients (Zn, Fe, Mn and B) are adequate. The ‘tal’ soils have high water-holding capacity (around 50%) due to the presence of high amount of finer fraction and develop cracks during summer (1 cm to more than 5 cm wide). The electrical conductivity of the soil is found to be low. The available nitrogen is medium in range (321.4–406.3 kg ha^−1^), available P_2_O_5_ is very low (2.5–14.6 kg ha^−1^) and available K_2_O is high in range (346–873 kg ha^−1^). The available micronutrient status is high except Zn, which are within critical limit (0.7–0.9 ppm). ‘Diara’ soil is known as “maize basket” of Bihar and ‘tal’ soil as “Pulse basket” of Bihar. Calcareous soil is light to heavy textured, contains more than 10% of calcium carbonate in silt and clay fraction, maximum limit of calcium carbonate content is 60% or more. Soils are fertile, containing available nitrogen (132–216 kg ha^−1^), available P_2_O_5_ (4.7–24.5 kg ha^−1^)and available K_2_O (206–315 kg ha^−1^) with 7.8–8.4 pH. Rice-based cropping system is common practice in this soil. Non-calcareous soils are light to medium textured, neutral to moderately acidic, moderately good to highly fertile and have poorly developed genetic horizon. Rice-based cropping system is prevalent in this soil.

**Table 1:** Physico-chemical characteristics of diara and tal soils

Character (range)	Soil type	
	Diara soil	Tal soil
pH	7.8–8.5	7.0–7.5
E.C. (dsm^−1^)	0.10–0.52	0.18–0.24
Organic carbon (%)	0.35–0.97	0.40–0.63
Water holding capacity (%)	29.4–32.0	46.3–50.4
Available nitrogen (kg/ha)	132.6–216.4	321.4–406.3
Available P_2_O_5_ (kg/ha)	4.7–24.5	2.5–14.6
Available K_2_O (kg/ha)	206–315	346–873
Available sulphur (ppm)	1.46–8.74	2.92–16.51
Available zinc (ppm)	0.66–1.32	0.68–0.90
Available boron (ppm)	1.08–2.65	0.4–1.03
Available Fe (ppm)	9.68–18.72	7.48–23.24
Available Mn (ppm)	8.90–12.54	9.02–14.98

### Soil sampling

Soil samples from fields under maize cultivation for 5 years (2005–2010) were collected from 0–15 cm depth. Loose soil was shaken off the roots and discarded, then the soil that adhered strongly to the roots was carefully brushed off and kept as rhizosphere soil. The samples were sieved (<2 mm) and stored at 4 °C until required for analysis, which was usually completed within 3 months. Eleven hundred and thirty-one samples were analysed for *Azospirillum* population enumeration.

### *Azospirillum* enumeration

Small test tube (10 ml) containing 5.0 ml NFb semi-solid nitrogen-free medium (Okon et al. 1977) was inoculated with serial dilution of rhizosphere soil suspension and enumeration was performed through MPN method (Doberneir 1995). Pellicle-forming ability and BTB (bromo thymol blue) test were followed as an indicator for positive ranking. After 3–5 days of incubation at 30 ± 2 °C, one loop of pellicle-forming culture was transferred into fresh semi-solid medium. Further, purification was done on NFb (Supplemented with 50 mg yeast extract l^−1^) and half strength DYGS medium agar plates (Eckert et al. 2001). Pellicle-forming cultures were maintained on half strength DYGS medium.

### Physiological characterization

Fifty *Azospirillum* isolates of different soils were characterized for growth, acetylene reduction assay (ARA) and IAA production to select efficient strains. Further, isolates were screened against pH and temperature tolerance range.

Growth of all fifty isolates was estimated by taking optical density at 620 nm (Tarrand et al. 1978) after incubation of *Azospirillum* isolates at 30 ± 2 °C for 48 h with shaking in BMS medium.

ARA of all fifty selected isolates was tested for nitrogenase activity. 10 ml of 48-h-old culture in NFb semi-solid medium (incubated at 32 ± 2 °C) was taken in sterilized glass vials of diameter 3.5 cm, capacity 35 ml with air tight rubber stopper under aseptic conditions. 3.5 ml air of each glass vials was replaced by an equal volume of acetylene using syringes and again incubated at 32 ± 2 °C for 90 min. 1 ml of gas phase was withdrawn from each bottle using syringes and injected into a gas chromatograph (Nucon-5700) using flame ionizing detector system with carrier gas nitrogen flow of 35 ml min^−1^ temperatures of oven, injector and detector were maintained at 85, 110 °C and temperature 120 °C, respectively, in a porapak-T column. The area of ethylene peak was recorded for each isolates and ARA was calculated with the help of ethylene standard curve (Jha et al. 2001).

The fifty isolates were further studied for Indole acetic acid production. Loop full of culture was incubated in 25 ml of Luria’s broth amended with 50 μg ml^−1^ tryptophan. The culture was incubated at 32 ± 2 °C on rotary shaker for 24 h and centrifuged at 10,000*g* for 15 min. Then 2 ml of the supernatant was taken in separate tubes and 2–3 drops of *O*-phosphoric acid were added with 4 ml of reagent containing 1 ml of 0.5 M FeCl_3_ in 50 ml of 85% HCIO_4_, again incubated for 25 min at room temperature and absorbance were recorded at 530 nm. Auxin was quantified with the help of IAA standard curve (Raja et al. 2006).

## Results and discussion

### Enumeration of *Azospirillum* in different soil types and agricultural practice

**Fig. 1:**
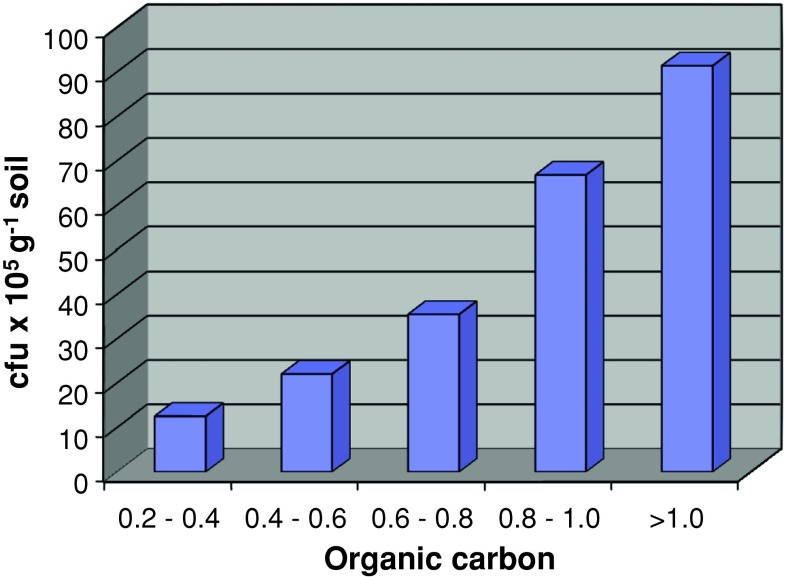
Native *Azospirillum* population under different organic carbon levels

Organic carbon status of the soil had a distinct impact on preponderance of *Azospirillum* in maize rhizosphere (Fig. 1). An increasing trend in population along with the increasing organic carbon concentration gradient was observed during experimentation. Minimum population of 12.5 × 10^5^ cfu g^−1^ soil at 0.2–0.4% organic carbon level was augmented to 91.4 × 10^5^ cfu g^−1^ soil at >1% organic carbon level. Intensive crop cultivation leads to a decline in soil organic matter (SOM) from the original pristine level and reaches an equilibrium value dictated by climate, rainfall and crop productivity. SOM content is the most important driver for the microbial activity and diversity (Wardle and Giller 1996). Our result suggests that SOM content can affect the *Azospirillum* population. An increase trend was observed in the preponderance of *Azospirillum* with an increase in organic carbon level from 0.2 to 1.0%. Our finding corroborates with Bashan et al. *(*1995) who observed that organic matter had a definite role in the survival of *Azospirillum* strains.

**Fig. 2:**
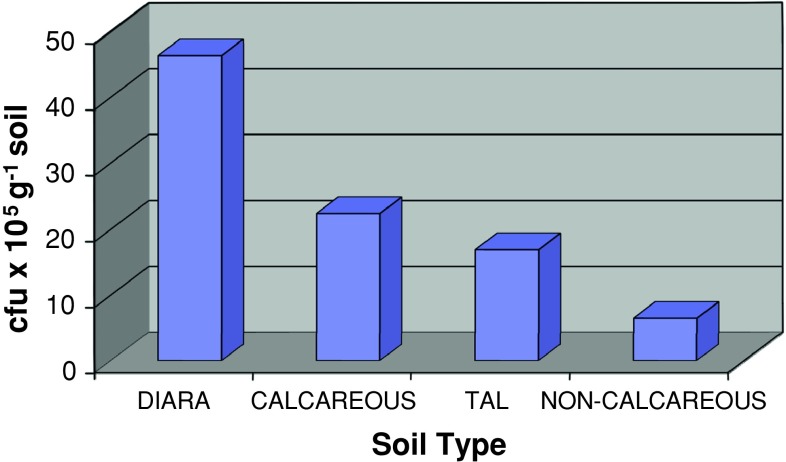
Native *Azospirillum* population under different soil types and land use history. Land use history: diara maize, tal: pulses, calcareous: rice/maize, non-calcareous: rice

The richness of *Azospirillum* community in maize rhizosphere was also influenced by soil types and land use history (Fig. 2). ‘Diara’ soil, a ‘maize basket of Bihar’ having history of maize cultivation since hundreds of years had highest population of 46.3 × 10^5^ cfu g^−1^ soil. The minimum population was observed in non-calcareous soil (6.5 × 10^5^ cfu g^−1^ soil) having history of rice cultivation. The ‘tal’ land, a pulse basket of Bihar, ranked third in terms of *Azospirillum* richness (16.9 × 10^5^ cfu g^−1^ soil). Such variation might be due to different physico-chemical factors and history of land use pattern. Introduction of maize crop in non-calcareous soil was much delayed than ‘diara’ soil. Soil types and land use history is an important component in dictating the microbial richness (Steenwerth et al. 2003; Buckely and Schimidt 2001; Schulter et al. 2004). In this study, we also assess the preponderance of *Azospirillum* in the maize rhizosphere of four soil types of different land use history using culture-based methods. Diara soil, a maize basket of Bihar having history of maize cultivation since hundreds of years had highest population of *Azospirillum* and non-calcareous soils, predominantly rice-growing areas had minimum population. The work of Smalla et al*.* (2001) has provided evidence that different plant species select different bacterial communities and that these plant-specific enrichment can be increased by repeated cultivation of the same plant species in the same field. The finding leads us to conclude that the plant host is the selective agent for predominant bacterial genera, which selects the partner from soil.

**Fig. 3:**
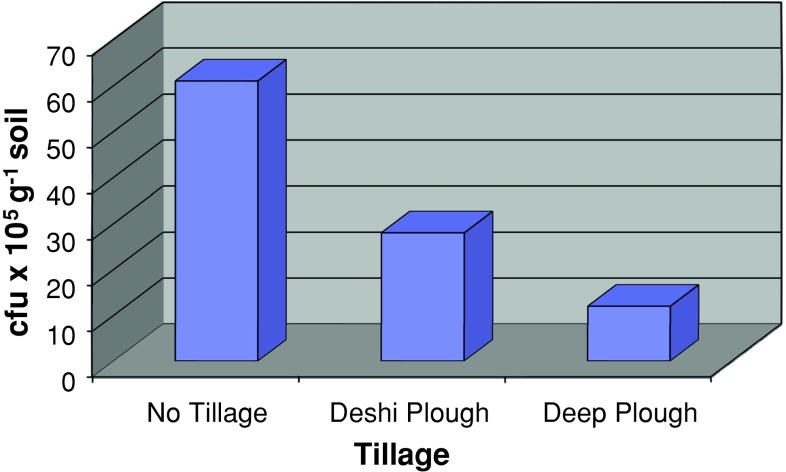
Native *Azospirillum* population under different tillage system

The practice of sowing maize by inserting maize seed through thumb or local driller or chisel (no till) in field after receding flood water is still practiced in some areas of ‘diara’ land and few pockets of other land types. Although, deep ploughing by tractor is a common practice in modern farming but many poor farmers still practice shallow tillage using country plough for maize cultivation. Such different tillage practices had great impact on population of *Azospirillum* in maize rhizosphere. No tillage encouraged maximum population of *Azospirillum* (61 × 10^5^ cfu g^−1^ soil) as compared to that of deep ploughing (12 × 10^5^ cfu g^−1^ soil) (Fig. 3). Soil disturbance by tillage can influence the structure and richness of microbial communities in the rhizosphere (Lupwayi et al. 1998; Giller 1996). Such disturbance may result in the reduction of microbial diversity due to desiccation, mechanical destruction and disruption of access to food resources. The numerical strength of *Azospirillum* in the present investigation decreased in deep tillage by tractor. Highest population was in the maize rhizosphere having no tillage practices. These results are consistent with those reported for other microbes by Hassink et al. (1991); Wander et al. (1995); Lupwayi et al. (1998). Apart from reducing the physical disturbance of the soil, no tillage leaves crop residues from the preceding years growth at the soil surface. In crop rotation under no tillage, the litter from several crops in preceding years is likely to result in a greater variety of substrate than deep tillage where litter does not accumulate.

**Fig. 4:**
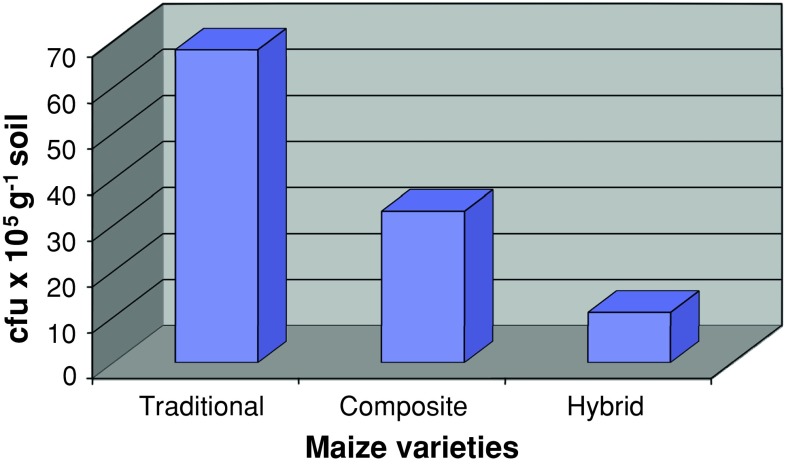
Native *Azospirillum* population under different maize varieties

Maize genotypes varied in terms of rhizosphere population of native *Azospirillum* to a great extent (Fig. 4). Traditional varieties harboured maximum *Azospirillum* population (68 × 10^5^ cfu g^−1^ soil) followed by that of composite maize (Laxmi, Vijaya, Swan) varieties (33 × 10^5^ cfu g^−1^ soil) and least in hybrid maize (Priya, Ganga-2, Kargil) (11 × 10^5^). This information is important in plant improvement programme and varietal planning. Maize genotypes had a pronounced effect on the preponderance of *Azospirillum* in its rhizosphere. Traditional or local cultivar of maize had highest *Azospirillum* population followed by composite maize cultivar. The population was decreased drastically in hybrid maize or modern cultivar. In fact, the development of modern hybrid varieties was based on its evaluation in standardized, high fertility systems with an emphasis on yield. In such system, beneficial interaction between plants and associated soil microorganisms made obsolete by the provision of nutrients in high quality and in readily available plant form. Furthermore, under such conditions, rhizosphere microbial communities are faced with an environment that differs substantially from the one in which plant microbial interaction originally evolved (Drinkwater and Snapp 2007). Thus, it may be difficult to select cultivars for low input agriculture form the elite germplasm pool of current cultivars. The reintroduction of genes regulating such adaptive traits from local cultivars into the gene pool of modern varieties may represent the most promising means to improve nutrient supply system through microbial approach.

**Fig. 5:**
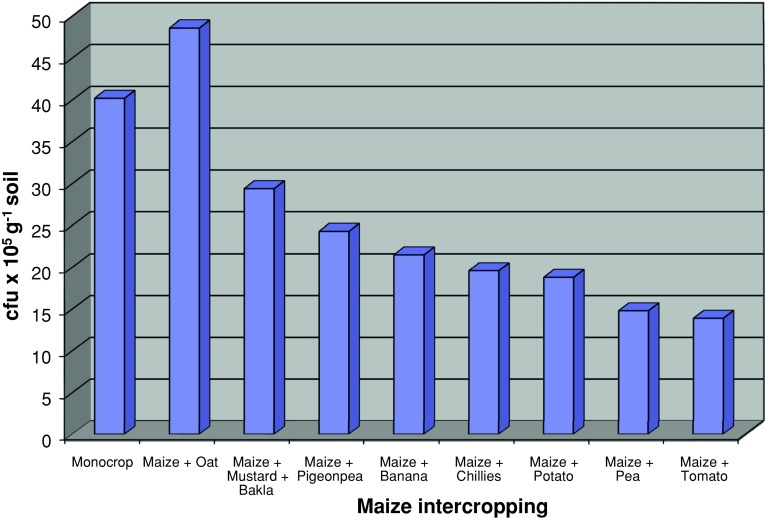
Native *Azospirillum* population under different monocrop and intercrop maize

*Azospirillum* population in monocropped and intercropped maize (cv. Laxmi) rhizosphere was monitored. Monocropped maize had more *Azospirillum* population (40.4 × 10^5^ cfu g^−1^ soil) than intercropped maize with pulse, vegetables and fruits (Fig. 5). However, intercropping of maize with cereals (oat) resulted in maximum population which was more than that of monocropping (48.6 × 10^5^ cfu g^−1^ soil). Probably higher root density (both of maize and oat having fibrous root system) per unit soil area, provided more proliferation surface (rhizoplane) for *Azospirillum.*

### Selection of promising strain

**Fig. 6:**
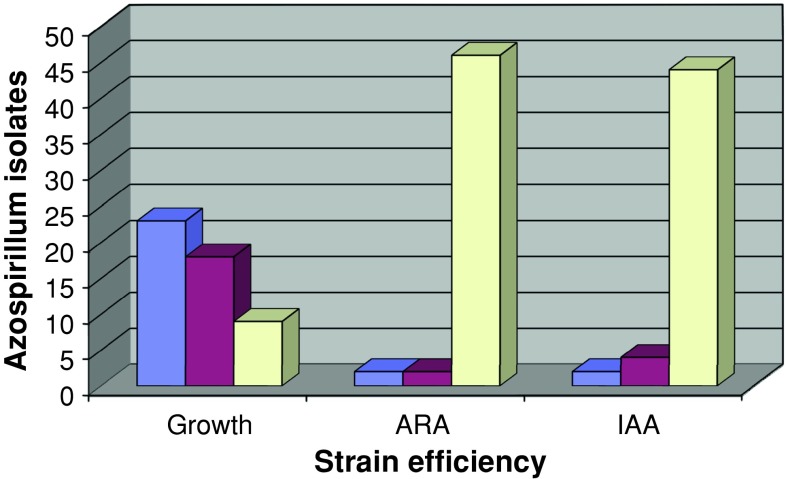
Evaluations of *Azospirillum* isolates for growth, ARA activities and IAA producing ability. *Grey square* excellent *black square* good *open square* poor. Growth: excellent (O.D. > 1.5), good (O.D. 1.0–1.5), poor (O.D. < 1.0). ARA (nmol C_2_H_4_ h^−1^ ml^−1^): excellent (>200), good (100–150), poor (<15). IAA (μg ml^−1^): excellent (>19.0), good (9.0–19.0), poor (<9.0)

Fifty *Azospirillum* isolates were evaluated for growth, ARA and IAA production. These isolates had its origin from all types of soil viz*.* ‘Diara’ soil (18 isolates, RAZ d series), ‘Tal’ soils (6 isolates, RAZ-t series), calcareous soils (12 isolates, RAZ-c series) and non-calcareous soils (14 isolates, RAZ-nc series). A summarized view of these evaluations is shown in Fig. 6. All the isolates are grouped into three categories that is excellent, good and poor, based on targeted characters of growth, nitrogen-fixing ability and IAA production. Forty-six percent isolates were excellent in growth (O.D. > 1.5) and only 36% were poor in growth (O.D. < 1.0).). Similarly, isolates were also grouped as excellent nitrogen fixer (>200 nmol C_2_H_4_ h^−1^ ml^−1^ culture) and good nitrogen fixer (100–150 nmol C_2_H_4_ h^−1^ ml^−1^ culture). However, 92% strains exhibited very poor ARA activity (<15 nmol C_2_H_4_ h^−1^ ml^−1^ culture). All the fifty *Azospirillum* isolates exhibited IAA production as indicated by the intensity of pink colour although the amount varied a lot depending on origin and nature of the isolates. Four percent isolates were excellent in IAA production (>19 μg ml^−1^), 8% were good in IAA production (9–19 μg ml^−1^) and 88% were poor in IAA production (<9 μg ml^−1^). Among all the isolates, three promising strains were identified as RAZ d_1_, RAZ-d9 and RAZ d_11_ on the basis of growth, ARA and IAA production. Higher numerical strength of *Azospirillum* strains in maize rhizosphere of Bihar soils might be due to prevalence of excellent and good growing strain but most of the strains were extremely poor in nitrogen-fixing ability (ARA) and plant growth promoting ability (IAA).Thus, such richness does not mean that there is no need of artificial inoculation of *Azospirillum* inoculants. In fact, *Azospirillum* biofertilization should be in universal mode rather than site-specific mode.

**Fig. 7:**
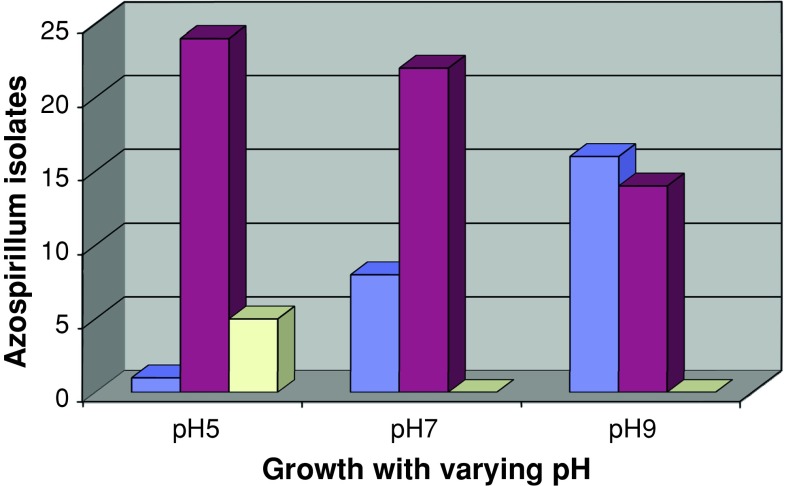
Growth behaviour of *Azospirillum* isolates under different pH level. *Grey square* excellent *black square* good *open square* poor. Growth: excellent (O.D. > 1.5), good (O.D. 1.0–1.5), poor (O.D. < 1.0)

**Fig. 8:**
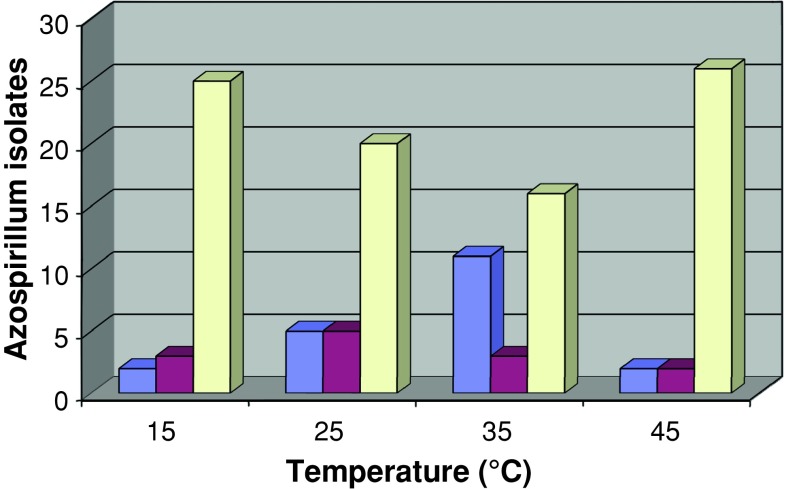
Growth behaviour of *Azospirillum* isolates under different temperature level. *Grey square* excellent *black square* good *open square* poor. Growth: excellent (O.D. > 1.5), good (O.D. 1.0–1.5), poor (O.D. < 1.0)

Thirty isolates were further exposed to stress conditions like pH and temperature variation. pH range selected for the experimentation was 5.0–9.0 and perusal of data in Fig. 7 indicated that pH changes did not affect the growth of *Azospirillum* isolates in appreciable manner. The strains which were having better growth in acidic range were RAZ-nc_4_ and RAZ-d13. Maximum growth at pH 9.0 was for RAZ-nc12. However, the isolates RAZ-nc_4_ and RAZ-d1 performed good in pH range of 5.0–9.0. Further, the temperature range selected for screening varied from 15 to 45 °C (Fig. 8). RAZ-d8 and RAZ-nc11 exhibited excellent growth at all the four range of selected temperature i.e. 15, 25, 35 and 45 °C, RAZ-d1 had maximum growth (O.D. 2.04) at 25 °C followed by 35 and 15 °C. It had minimum growth (0.D, 1.03) at 45 °C and 83% had poor growth at 15 °C. Most of the isolates preferred 25–35 °C for optimum growth. RAZ-nc11, RAZd_8_ and RAZ-d1 can be effectively used for both kharif and rabi maize.

Preponderance of the microorganisms plays an important role in sustaining the soil health and plant growth. The information generated in this work clearly indicated that Bihar soil is rich in *Azospirillum* population which varied from 1 million to 1 billon/g soil under diverse agricultural practices (tillage, crop varieties), soil types and organic matter level. However, the present study also indicated that 92% of the evaluated *Azospirillum* isolates (50 isolates) were poor in nitrogen-fixing ability and 88% were poor in IAA production. Such information suggests that *Azospirillum* biofertilization should be in universal mode rather than site-specific mode through introduction of efficient or promising strains of *Azospirillum* for biofertilization programme. Various studies indicated that the magnitude of *Azospirillum* responses varied with location, seasons, product quality etc. (Bashan et al*.*, 2004). Further, chances of survival of aliens strain in poor than autochthonous strain (Bashan et al*.*, 1995). Thus, selection of region specific promising strains is the prerequisite for deriving maximum benefit from *Azospirillum*. Such selection should not be only based on the targeted character but also on stress tolerance. Since in Bihar, maize is cultivated throughout the year so there is an immediate need of such *Azospirillum* strain which can enjoy the broader range of temperature. In the present investigation, such promising strain was identified with tolerance to broader range of temperature and pH.

## Conclusion

No tillage, traditional maize cultivar, land use history and soil organic matter supported *Azospirillum* prepondrance in the rhizosphere of maize. However, such richness does not mean that there is no need of biofertilization as majority of the *Azospirillum* isolates were poor in targeted character. In fact, biofertilization should be in universal mode rather than site-specific mode. The information generated in the project from complex natural communities such as farmer’s agricultural field may be more relevant than the information generated under green house or in simulated condition.
